# Mindfulness meditation and improvement in depressive symptoms among Spanish- and English speaking adults: A randomized, controlled, comparative efficacy trial

**DOI:** 10.1371/journal.pone.0219425

**Published:** 2019-07-05

**Authors:** Eric Lopez-Maya, Richard Olmstead, Michael R. Irwin

**Affiliations:** Mindful Awareness Research Center and Cousins Center for Psychoneuroimmunology, Semel Institute for Neuroscience and Human Behavior, Department of Psychiatry and Biobehavioral Sciences, Geffen School of Medicine, University of California, Los Angeles, California, United States of America; Yale School of Medicine, UNITED STATES

## Abstract

**Objective:**

Latino immigrants experience acculturative stress and increased depression risk. Mindfulness meditation improves depressive symptoms, yet the vast majority of research has focused on English speaking populations.

**Methods:**

In this randomized clinical trial with 2 parallel treatment groups, adults with moderate levels of perceived stress (n = 76) were recruited from the Los Angeles community from October 2015 to March 2016, stratified into Spanish- (n = 36) and English speaking (n = 40) language groups, and randomized for 6 weeks of treatment with standardized mindful awareness practices (MAPs) or health education (HE). Main outcome measure was depressive symptoms, measured by the Beck Depression Inventory.

**Results:**

Using an intent-to-treat analysis, the primary outcome, depressive symptoms as indexed by the Beck Depression Inventory, showed greater improvement in MAPs vs. HE, with a between-group post-intervention mean difference of -2.2 (95% CI -4.4 – -0.07) and effect size of 0.28; similar effect sizes were found in the the Spanish- (0.29) and English speaking (0.30) groups. MAPs showed significant improvement relative to HE on secondary outcome of mindfulness with between group difference of 10.7 (95% CI4.5–16.9), but not perceived stress.

**Conclusion:**

The comparable efficacy of Spanish and English formats of mindfulness meditation in improving depressive symptoms suggests that this community based intervention may mitigate depression risk in Latino adults who are experiencing social adversity.

**Trial registration:**

ClinicalTrials.gov NCT03545074.

## Introduction

Stressful life events, especially major life events, substantially increase risk for depressive symptoms and major depressive disorder [1,2], with up to 80% of major depressive episodes in the general population being precipitated by such stress[3]. Among the various types of major life events, adjustment to a non-native culture (i.e., acculturative stress) can cause substantial cognitive upheaval and disruption to a person’s goals, plans, and aspirations, resulting in psychological distress and depressive symptoms, as well as the onset of a major depressive disorder[4,5]. Indeed, within the United States, increasing evidence indicates that a high prevalence of Latino and Spanish-speaking populations are experiencing acculturative stress, and that such acculturation is linked to depressive symptoms, decreases in quality of life, and higher rates of depression[6–9]. Addressing moderate depressive symptoms using community-accessible programs is a promising public health approach to mitigate the risk of depression and other adverse mental health outcomes, yet there is a striking absence of controlled trial research that has targeted Spanish speaking populations.

Mindfulness-based interventions (MBIs) hold the potential to possibly meet the needs for a scalable community-accessible treatment that improves depressive symptoms in adults experiencing stress. People use meditation to mitigate the effects of stress, and meta-analytic findings demonstrate that mindfulness meditation programs show improvements in depressive symptoms, when compared with nonspecific active controls[10]. Furthermore, we have previously found that a standardized mindfulness curriculum, mindful awareness practices (MAPs), reduces depressive symptoms and improves sleep in community dwelling older adults[11]. MAPs trains one in the systematic practice of attending to moment-by-moment experiences, thoughts, and emotions from a nonjudgmental perspective[12], similar to Mindfulness Based Stress Reduction (MBSR). However, in contrast to MBSR, MAPs is more accessible as it does require a day-long retreat or Hatha yoga; these two components of MBSR might substantially limit dissemination and adherence in Spanish speaking populations experiencing acculturative stress.

The vast majority of research on the efficacy of mindfulness interventions on depressive symptoms has been performed with English speaking participants, largely ignoring language and possible culture differences[10]. Whereas culturally relevant mindfulness interventions have been developed for African-American, Native American and even Latino communities in Latin America [13], research focused on Latino communities in the United States is limited[14]. To our knowledge, no prior study has evaluated the comparative efficacy of Spanish vs. English formats of a mindfulness program on depressive symptoms in these two language groups in the United States.

The primary objectives of the current study are to determine whether MAPs vs. Health Education (HE) improves depressive symptoms in adults with moderate levels of perceived stress, and whether the efficacy of MAPs vs. HE is comparable between Spanish-speaking and English-speaking formats. Among adults who are report moderate levels of perceived stress, we hypothesized that MAPs would show similar effects on improving depressive symptoms using Spanish-speaking and English speaking formats in two respective language groups. Secondary outcomes were mindfulness and perceived stress.

## Methods

### Trial design

Ethical approval for the trial was obtained from the UCLA Institutional Review Board (IRB) on October 3^rd^, 2015 with subject recruitment conducted from October 10^th^ 2015 to March 31st 2016 with completion of follow-up on June 30, 2016. Trial design was posted in Clinical Trials.gov (NCT03545074) on June 4, 2018; delay in trial registration until after completion of the study was due to administrative oversight. The authors confirm that all ongoing and related trials for this intervention are registered. All subjects provided written consent and all procedures were approved by the UCLA Human Subjects Protection Committee (HSPC). In addition, all methods were performed in accordance with the relevant guidelines and regulations approved by the UCLA HSPC. including a statement in the methods section to this effect.

The study is a single-masked (rater), single-site, parallel-group, randomized controlled comparative efficacy trial of MAPs versus HE on depressive symptoms, and secondary outcomes of mindfulness and psychological stress, in moderately stressed adults who are either English- or Spanish speaking.

After recruitment by advertisement, telephone screening, informed consent, and completion of questionnaires and interviews, participants were stratified into English-speaking or Spanish-speaking groups and then randomly assigned to MAPs or HE for 6 weeks. Assessments were re-administered at post-intervention.

### Study participants

The eligibility criteria were adults between 18 and 60 years old who expressed interest in learning tools for stress management. English speakers were not Latinos, as assessed during the interview. Fluency in English or Spanish was assessed by self-report, and verified by interview. Given that depressive symptoms was the primary outcome, subjects were eligible if they reported psychological distress as scored by a 9 or more on the Perceived Stress Scale [15]. Severity of depressive symptoms was not used as a screening criteria as this might unmask the hypothesis of the study focused on improvement in self-reported depressive symptoms. Finally, participants were excluded if they were taking psychotropic medications on a regular basis, routinely using pain medications, and taking other medications that might affect the immune system due to evidence that such medications can alter depressive symptoms[16,17].

### Interventions

Mindful Awareness Practices for Daily Living Program (MAPs). The MAPs is a weekly 2-hour, 6-session group-based course in mindfulness meditation that is available to take in person or online (http://marc.ucla.edu), as previously described[11]. A certified teacher with more than 20 years of mindfulness practice developed this validated and curriculum based mindfulness program. Briefly, session based learning is focused on mindfulness exercises including mindful sitting meditation, mindful eating, appreciation meditation, friendly or loving-kindness meditation, mindful walking, and mindful movement. In each session, participants engaged in 30 minutes of mindful experiential practice, in addition to the teacher-delivered didactic material and group discussion. Participants were also provided with a book on mindfulness accompanied by a guided meditation compact disc [18]. Mindfulness practice homework began with 5 minutes daily and then progressively advanced to 20 minutes daily by session 6 [11]. In this regard, MAPs is similar to other Mindfulness-Based Interventions, such as MBSR or MBCT in terms of the structure of the program, which includes equivalent practice components (both formal and informal), didactic elements and group process features, yet with the advantage of making the MAPs more accessible than MBSR, since MAPs does not include an all-day retreat and Yoga practices. The curriculum based research manual was translated from English to Spanish by E.L. who has more than 15 years of experience teaching mindfulness in different settings, and a certified teacher with more than 10 years’ experience delivering the MAPs in English- or Spanish-speaking formats.

Health Education Program (HE) The HE is a weekly 2-hour, 6 session course aimed at knowledge acquisition in subjects related to health care in general. Similar interventions have been described elsewhere [11,19]. A trained health educator provided videos and didactic presentations on topics such as: stress, sleep hygiene, diet and nutrition, sexuality, mental health and substance abuse. The health education condition resembled the MAPs intervention in terms of duration, group format and support, attention and participant expectancy regarding health benefits. Homework included practicing health habits and weekly reading, with in-class group discussion to match the homework assigned in the MAPs group.

### Treatment fidelity

Therapists were experienced and trained in one modality but not in the other. Another therapist who had more than 15 years of experience in either MAPs or HE provided weekly supervision, and evaluated treatment integrity.

### Primary outcome

The primary outcome measure was severity of depressive symptoms as assessed by the Beck Depression Inventory (BDI) [20], with evidence that the BDI reliably evaluates depressive symptoms in non-psychiatric samples [21,22], as well as those who are Spanish speaking [23].The selection of the primary outcome was made because the study was advertised as a research study to improve perceived stress and therefore participants were partially blinded to the hypothesis, which focused on severity of depressive symptoms.

### Secondary outcomes

Secondary outcomes included self-reported levels of mindfulness as measured by the Five-Facet Mindfulness Questionnaire,(FFMQ) [24] and perceived stress, as measured by the Perceived Stress Scale [15].

### Sample size

Power was estimated in Gpower (http://www.gpower.hhu.de/en.html) and is based on prior meta-analytic findings [10], and on our prior findings that have examined the effects of MAPs and HE on depressive symptoms as measured by the BDI [11]. Indeed, the observed effect size in the meta-analysis (d = 0.3)[10] was balanced with the observed effect in our prior study (d = 0.89) [11] to achieve a midpoint of these two effect sizes (d = 0.60). The test-retest reliability of the BDI was conservatively estimated to be r = .60 but it is typically observed to be higher. Under these assumptions, an estimated sample size 36 is needed to achieve 80% power, even with possible drop-outs and possible additional variability due to the language factor. Hence, the overall sample for this study was 72, with 36 in each language group to test between treatment effects of difference at post-intervention.

### Randomization

Random assignment sequence was generated via a computerized random number generator in blocks of 7 to 10 participants in MAPs and HE (1:1) for English- and Spanish speaking groups by R.O., who did not view participant data before allocation. To maintain concealment, no research staff had access to allocation sequence, which was recorded on sequentially numbered, opaque, and sealed envelopes.

### Blinding

The study was advertised as a research study to evaluate whether one or another treatment would improve perceived stress, and participants remained blind to hypotheses and the content of the other treatment group through study duration. Use of a modified blind-to-treatment protocol or partial blinding, which is thought to reduce selection bias that is frequently associated with trials of behavioral interventions. Thus, depression, as opposed to perceived stress, was chosen as the primary outcome variable to reduce the aforementioned bias. Investigators and outcome assessors were blinded to allocation.

### Statistical analyses

Between treatment difference in change in mean BDI at post-intervention was the primary outcome in the intent-to-treat population. Analyses were performed in SPSS, version 21 (IBM Corporation). Treatment effects, covarying for pre-intervention values, were tested using a mixed multi-level model approach for primary and secondary outcomes. Data from all randomly assigned participants were included. Missing data were less than 10% and were imputed using the expectation maximization method. The mixed model approach generated unbiased estimates under the assumption that data are missing completely at random and the missing completely at random assumption was tested. Estimated mean differences at post-intervention were tested by treatment (MAPs vs HE) and also by language group (English vs. Spanish). Treatment effect sizes (Cohen d with Hedges bias correction for small sample size) with their 95% CIs are provided for the total sample, and for each language group.

Differences in treatment effect size (MAPs vs HE) for the primary outcome, BDI, were compared for equivalence by language groups using methods proposed by Steiger[25]. Briefly, this test of equivalence constructs a confidence interval (CI) and then compares it to a "range of triviality" (ROT). In this case, the ROT was set at a Cohen's d difference between -0.1 and 0.1. To determine equivalence, we examined whether the CI falls fully within the ROT, which statistically supports equivalence. Furthermore, if the CI falls completely outside the ROT, then equivalence is rejected. However, when the CI crosses the ROT, equivalence is neither confirmed nor rejected; the test is inconclusive.

## Results

### Pre-intervention characteristics of participants

**Fig 1:**
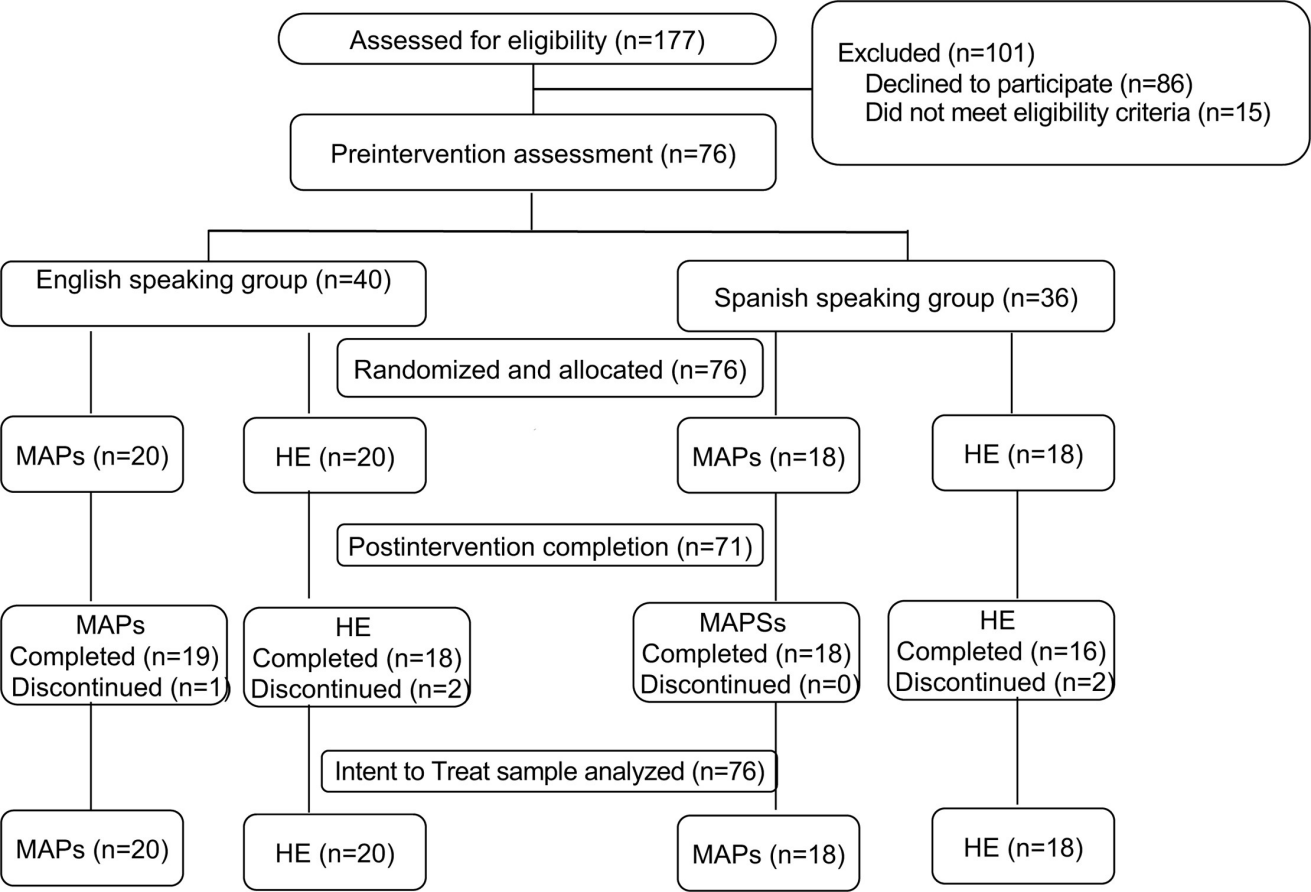
Consolidated standards of reporting trials flow diagram of the single-site, parallel-group randomized clinical trial of MAPs compared with HE for depressive symptoms in English- and Spanish speaking adults.

Table 1 lists the pre-intervention characteristics of the two treatment conditions stratified by English- and Spanish speaking groups. In the total sample, as well as the two language groups, there were no pre-intervention differences between the MAPs and HE treatment groups in demographic characteristics, depressive symptoms, resting mindfulness states, or perceived stress (all P’s>0.12). Fig 1 shows participant flow through enrollment, exclusion, randomization, allocation and analysis phases of the trial. Of the 76 subjects who were randomized and allocated to treatment, 71 completed post-intervention assessments, with 1 subject dropping from MAPs and 2 subjects from HE in the English-speaking group, and 2 subjects dropping from HE in the Spanish-speaking group. The demographic and clinical characteristics of those who dropped from the intervention did not differ from the overall sample (all P’s>0.23). There were no reported adverse events related to participation in the study.

**Table 1:** Preintervention demographic and clinical characteristics in the total sample and in the English- and Spanish speaking groups^a^.

	Preintervention		
	MAPs	HE	
			Group Difference; P value
**Age, mean (SD), y**			
**Total sample^a^**	43.4 (16.4)	42.2 (14.4)	.75
**English-speaking group^b^**	44.2 (17.7)	43.6 (14.7)	.91
**Spanish-speaking group^c^**	42.5 (15.2)	40.5 (14.3)	.72
**Female sex, No. (%)**			
**Total sample**	31 (81.6)	29 (76.3)	.78
**English-speaking group**	15 (75.0)	16 (80.0)	1.00
**Spanish-speaking group**	16 (88.9)	13 (72.2)	.41
**Educational level, mean (SD), y**			
**Total sample**	16.1 (3.4)	15.9 (4.2)	.88
**English-speaking group**	15.7 (4.2)	16.0 (4.7)	.81
**Spanish-speaking group**	16.6 (2.3)	15.8 (4.0)	.52
**Married, No. (%)**			
**Total sample**	16 (43.2)	11 (29.7)	.34
**English-speaking group**	7 (35.0)	7 (35.0)	1.00
**Spanish-speaking group**	9 (52.9)	4 (23.5)	.16
**Employed, No.(%)**			
**Total sample**	21 (56.8)	27 (71.1)	.24
**English-speaking group**	8 (40.0)	14 (70.0)	.12
**Spanish-speaking group**	13 (76.5)	13 (72.2)	1.00
**Beck Depression Inventory**			
**Total sample**	17.1 (8.3)	15.9 (7.6)	.52
**English-speaking group**	19.6 (7.5)	17.4 (7.7)	.37
**Spanish-speaking group**	14.3 (8.4)	14.2 (7.3)	.99
**Five Facet Mindfulness Questionnaire**			
**Total sample**	114.6 (20.7)	119.2 (19.6)	.33
**English-speaking group**	108.2 (17.7)	117.0 (20.3)	.16
**Spanish-speaking group**	121.6 (21.9)	121.5 (19.0)	.99
**Perceived Stress Scale**			
**Total sample**	23.6 (5.5)	24.3 (5.8)	.51
**English-speaking group**	25.9 (5.5)	26.6 (6.8)	.71
**Spanish-speaking group**	20.8 (4.2)	21.8 (3.2)	.42

^a^Total sample N = 76

^b^English-speaking group N = 40

^c^Spanish speaking group N = 36

### Primary outcome

Primary and secondary outcome intent-to-treat analyses included all participants (N = 76) according to original treatment assignment regardless of program attendance or missing data. In the overall sample, MAPs showed greater improvement in severity of depressive symptoms as indexed by the BDI from pre- to post-intervention as compared to HE (between-treatment post-intervention mean difference: -2.2, 95% CI -4.4 –-0.07). (Table 2) English- and Spanish speaking groups did not differ at post-intervention, indicating that the two language groups changed similarly in the two conditions. In addition, effect sizes for post-intervention group differences were similar in the total sample, and in the two language groups. (Table 2) To evaluate equivalency, we found that the point estimates of the group difference in treatment effect sizes for BDI were all within the ROT. However, the test for equivalence confidence intervals extended beyond the range of triviality. Hence, equivalency was not completely rejected, nor was it statistically confirmed.

**Table 2:** Intent-to-Treat Model Estimates for Primary and Secondary Outcome Measures in the Total Sample and in the English- and Spanish Speaking Groups^a^.

	Preintervention (Mean SE)		Postintervention (Mean SE)		Value (95% CI)	Value (95% CI)	
	MAPs	HE	MAPs	HE	Postintervention Treatment	Postintervention Language	
Primary Outcome					Group Difference	Group Difference^b^	Effect Size^c^
**Beck Depression Inventory**							
**Total sample**	16.6 (0.8)	16.3 (0.8)	6.5 (0.8)*	8.7 (0.8)*	-2.2 (-4.4 to -0.07)	1.0 (-1.2 to 3.2)	.28
**English-speaking group**	17.4 (1.1)	16.7 (1.1)	7.0 (1.1)*	9.2 (1.1)*			.30
**Spanish-speaking group**	15.8 (1.1)	15.8 (1.1)	6.0 (1.1)*	8.2 (1.1)*			.29
**Secondary Outcomes**							
**Five Facet Mindfulness Questionnaire**							
**Total sample**	116.3 (2.2)	117.6 (2.2)	140.8 (2.2)*	130.1 (2.2)*	10.7 (4.5 to 16.9)	3.7 (-2.5 to 9.9)	.53
**English-speaking group**	114.2 (3.1)	116.9 (3.0)	142.0 (3.1)*	132.5 (3.0)*			.49
**Spanish-speaking group**	118.3 (3.2)	118.3 (3.2)	139.5 (3.2)*	127.6 (3.2)*			.59
**Perceived Stress Scale**							
**Total sample**	23.6 (0.7)	24.0 (0.7)	16.3 (0.7)*	17.6 (0.7)*	-1.3 (-3.3 to 0.7)	-2.5 (-4.6 to -0.5)	.23
**English-speaking group**	24.8 (1.0)	25.2 (1.0)	14.8 (1.0)*	16.6 (1.0)*			.29
**Spanish-speaking group**	22.4 (1.0)	22.8 (1.0)	17.8 (1.0)*	18.7 (1.0)*			.23

^a^Analyses covarying for baseline

^b^English minus Spanish score

^c^Effect size for postintervention treatment group difference

*Significant P<0.05 change from preintervention to postintervention

### Secondary outcomes

Table 2 also lists summary results for the secondary outcomes. In the overall sample, MAPs show robust increases in mindfulness as indexed by the Five Facet Mindfulness Questionnaire from pre- to post-intervention as compared to HE (between-treatment post-intervention mean difference: 10.7, 95% CI 4.5–16.9), with similar effect sizes for post-intervention treatment differences in the total sample, and in the two language groups. (Table 2). However, both MAPs and HE yielded similar levels of improvement in the Perceived Stress Scale, in which between treatment post-intervention mean differences were statistically significant.

## Discussion

This randomized, controlled, partially blinded, comparative efficacy trial examined the effects of MAPs vs. HE on severity of depressive symptoms in adults with moderate levels of perceived stress, and evaluated whether the efficacy of MAPs vs. HE is comparable between Spanish-speaking and English-speaking formats. As compared to HE, MAPs yielded greater improvements in depressive symptoms at post-intervention, in which effect sizes were comparable between the Spanish-speaking and English speaking formats in the two respective language groups. Indeed, during the course of 6 weeks of treatment, the MAPs effect size for improvement in depressive symptoms was 0.28 in the overall sample, with similar effect sizes in the Spanish-speaking (0.29) and English-speaking groups (0.30). Whereas these results indicated that equivalence was not rejected, the differences between the language groups extended beyond and range of triviality. Hence, despite similar effect sizes, equivalence was not statistically confirmed.

MAPs yielded also robust increases in mindfulness, in which effect sizes were also comparable in the Spanish- and English speaking groups. In this sample who were experiencing moderate levels of perceived stress, modest improvements in perceived stress were found for both MAPs and HE conditions, yielding similar benefits in both MAPs and HE conditions. Together these findings indicate that MAPs differentially alters psychological responses to perceived stress with improvements in depressive symptoms, even in the presence of ongoing perceived stress. Moreover, because the benefits of MAPS on depressive symptoms were as robust in the Spanish-speaking group as compared to those who were English speaking group, delivery of this Spanish language format of mindfulness meditation to Latino populations has potential to serve as a community-accessible program to mitigate the risk of depression, with implications for depression in response to acculturation stress among immigrant Latino adults [26].

The effect size of MAPs on depressive symptoms, the primary outcome, is comparable to other mindfulness meditation programs, in which meta-analytic effect size estimates range from 0.23 to 0.30 in the improvement of for reducing depressive symptoms [10]. Importantly, these effect sizes are comparable to what has been found following the use of an antidepressant in a primary care population [27]. For example, Fournier et al. reported that for patients with mild to moderate depressive symptoms, the effect size for antidepressants was only 0.11 (95% CI, −0.18 to 0.41) compared with placebo [27]. Because MAPs resulted in similar, although not statistically equivalent effects. in the Spanish speaking group, as compared to the English speaking group, this mindfulness program may be especially relevant for Latino populations who are experiencing social adversity, yet have limited access to traditional mental health services to treat depressive symptoms and improve mental health outcomes. Finally, whereas others have reported that mindfulness improves psychological stress in Spanish-speaking populations [28,29], this study is innovative by showing that benefits of mindfulness are similar to English or Spanish formats, which supports the ecological validity of mindfulness interventions in terms of language and cultural diversity.

The benefits of mindfulness practices appear to extend beyond improving mental health outcomes, as we and others have found the mindfulness based interventions including mindfulness meditation, tai chi, and yoga, have robust effects on inflammatory biology [30–34]. Inflammation is found to be increased in persons experiencing social adversity, and because inflammation represents a key hallmark of chronic disease risk and biological aging [35–37], changes in inflammatory biology provide a mechanistic understanding of why stressed persons show increased risk for adverse physical and mental health outcomes [1,38]. Indeed, mindfulness based interventions have been found to have robust effects to induce a down-regulation of inflammatory gene expression even after relatively short (6 week) interventions [32,34], with further evidence that monocyte production of proinflammatory cytokines in response to an inflammatory challenge is reduced as early as 8 weeks after onset of a mindfulness intervention [33,39]. Moreover, mindfulness based interventions can reduce circulating markers of inflammation such as C-reactive protein [40], a predictor of chronic disease risk including diabetes and cardiovascular disease [38], although it appears that these effects take longer to emerge (i.e., months) and may require more intensive practice of the treatment [31,32,41,42]. Nevertheless, because mindfulness can be readily integrated into one’s daily, this practice has the potential for long term benefit on physical and mental health outcome, especially for Latino populations who show higher levels of inflammation, along with diabetes and cardiovascular disease [42,43]. This study is characterized by several strengths, including defined eligibility criteria including the presence of at least moderate levels of psychological or perceived stress; use of a modified blinded-to-treatment protocol that was intended to reduce selection bias; random assignment; manualized interventions; matching of treatment exposure time and attention; and low rates of drop-outs in the two condition and language groups. Additionally, the two language groups were comparable on demographic and clinical characteristics.

This study had several limitations. There was a lack of treatment effect on perceived stress, which is not consistent with prior meta-analytic findings studies. Indeed, both treatments, MAPs and HE, showed decreases in perceived stress from pre- to post-intervention, which indicates that the active comparator control condition, health education, was also effective in providing information that helped participants manage psychological stress, even though such improvements in perceptions of stress did not mitigate depressive symptoms. However, the absence of difference between MAPs and HE on this outcome does not necessarily mean the MAPs was not effective, but rather MAPs was not superior to HE. Secondly, of the subjects who underwent screening eligibility, about half declined to participate, which might suggest a selection bias as to who will participate in a mindfulness program, or possibly health education, training program. Employing Latino recruiters at a community level might have mitigated the high rate of refusal. Third, assessments were limited to pre- and post-intervention, and it is not known whether the benefit of mindfulness to improve depressive symptoms show equivalent durability between the two language groups in the long term. Fourth, no measure of acculturative stress, years in the United States or trauma related to crossing the border was obtained. Finally, religious practices may differ between the two groups, and this was not assessed.

These findings demonstrate that MAPs is superior to an active comparator control, HE, in improving depressive symptoms in persons who are experiencing moderate levels of perceived stress. Further, the efficacy of MAPs was similar, though not statistically equivalent in the two language groups, which suggest that a Spanish format of MAPs is effective, similar to the English format, in promoting mindfulness and improving depressive symptoms. The scalability, relative low cost, and accessibility of mindfulness programs has the potential to address the need for community based interventions to be delivered to Spanish-speaking communities in the U.S. and around the world to lessen the risk of depression and possibly other adverse mental health outcomes.

## Supporting information

S1 Consort Checklist Consort checklist. (PDF)

S1 File Protocol. (DOCX)
