# Supplementary material for: Mindfulness meditation and improvement in depressive symptoms among Spanish- and English speaking adults: A randomized, controlled, comparative efficacy trial
Source: PLoS One. 2019 Jul 5;14(7):e0219425. doi: 10.1371/journal.pone.0219425 (PMC6611613; doi:10.1371/journal.pone.0219425)
Supplement: S1 File — (DOCX) [file pone.0219425.s002.docx]

**Protocol**

**Mindfulness Meditation and Improvement in Depressive Symptoms**

**Among Spanish- and English Speaking Adults:**

**A Randomized, Controlled, Comparative Efficacy Trial**

Eric Lopez-Maya, Richard Olmstead, Michael R. Irwin, M.D.

**BACKGROUND**

Stressful life events, especially major life events, substantially increase risk for depressive symptoms and major depressive disorder^1,2^, with up to 80% of major depressive episodes in the general population being precipitated by such stress^3^. Among the various types of major life events, adjustment to a non-native culture (i.e., acculturative stress) can cause substantial cognitive upheaval and disruption to a person’s goals, plans, and aspirations, resulting in psychological distress and depressive symptoms, as well as the onset of a major depressive disorder^4,5^. Indeed, within the United States, increasing evidence indicates that a high prevalence of Latino and Spanish-speaking populations are experiencing acculturative stress, and that such acculturation is linked to depressive symptoms, decreases in quality of life, and higher rates of depression^6-9^. Addressing moderate depressive symptoms using community-accessible programs is a promising public health approach to mitigate the risk of depression and other adverse mental health outcomes, yet there is a striking absence of controlled trial research that has targeted Spanish speaking populations.

Mindfulness-based interventions (MBIs) hold the potential to possibly meet the needs for a scalable community-accessible treatment that improves depressive symptoms in adults experiencing stress. People use meditation to mitigate the effects of stress, and meta-analytic findings demonstrate that mindfulness meditation programs show improvements in depressive symptoms, when compared with nonspecific active controls^10^. Furthermore, we have previously found that a standardized mindfulness curriculum, mindful awareness practices (MAPs), reduces depressive symptoms and improves sleep in community dwelling older adults^11^. MAPs trains one in the systematic practice of attending to moment-by-moment experiences, thoughts, and emotions from a nonjudgmental perspective^12^, similar to Mindfulness Based Stress Reduction (MBSR). However, in contrast to MBSR, MAPs is more accessible by not requiring a day-long retreat or Hatha yoga.

The vast majority of research on the efficacy of mindfulness interventions on depressive symptoms has been performed with English speaking participants, largely ignoring language and possible culture differences^10^. Whereas culturally relevant mindfulness interventions have been developed for African-American and Native American communities, research focused on Latino communities is limited^13^, and no prior study has evaluated the comparative efficacy of Spanish vs. English formats of a mindfulness program on depressive symptoms in these two language groups.

The primary objectives of the current study will be to determine whether MAPs vs. HE improves depressive symptoms in adults with moderate levels of perceived stress, and whether the efficacy of MAPs vs. HE is comparable between Spanish-speaking and English-speaking formats. Among adults who are report moderate levels of perceived stress, we hypothesize that MAPs would show similar effects on improving depressive symptoms using Spanish-speaking and English speaking formats in two respective language groups. Secondary outcomes were mindfulness and perceived stress.

**METHODS**

*Trial Design*

Trial design will be registered in Clinical Trials.gov and ethical approval wil be obtained from the UCLA Institutional Review Board (IRB). All subjects will provide written consent and all procedures will be approved by the UCLA Human Subjects Protection Committee (HSPC). In addition, all methods will be performed in accordance with the relevant guidelines and regulations approved by the UCLA HSPC. including a statement in the methods section to this effec

The study will be a single-masked (rater), single-site, parallel-group, randomized controlled comparative efficacy trial of MAPs versus HE on depressive symptoms, and secondary outcomes of mindfulness and psychological stress, in moderately stressed adults who are either English- or Spanish speaking.

After recruitment by advertisement, telephone screening, informed consent, and completion of questionnaires and interviews, participants will be stratified into English-speaking or Spanish-speaking groups and then randomly assigned to MAPs or HE for 6 weeks. Assessments will re-administered at post-intervention.

*Study Participants*

Recruitment will conducted from beginning October 2015.

The eligibility criteria will be:

1. Adults between 18 and 60 years old who express interest in learning tools for stress management.

2. Fluency in English or Spanish as assessed by self-report, and verified by interview.

3. Given that depressive symptoms will be the primary outcome, subjects are eligible if they report psychological distress as scored by a 9 or more on the Perceived Stress Scale^14^. We will not use severity of depressive symptoms as a screening criteria as this might unmask the hypothesis of the study focused on improvement in self-reported depressive symptoms.

4. Participants will be excluded if they were taking psychotropic medications on a regular basis, routinely using pain medications, and taking other medications that might affect the immune system due to evidence that such medications can alter depressive symptoms^15,16^.

*Interventions*

Mindful Awareness Practices for Daily Living Program (MAPs)

The MAPs is a weekly 2-hour, 6-session group-based course in mindfulness meditation that is available to take in person or online (<http://marc.ucla.edu)>, as previously described^11^. A certified teacher with more than 20 years of mindfulness practice developed this validated and curriculum based mindfulness program. Briefly, session based learning will be focused on mindfulness exercises including mindful sitting meditation, mindful eating, appreciation meditation, friendly or loving-kindness meditation, mindful walking, and mindful movement. In each session, participants engaged in 30 minutes of mindful experiential practice, in addition to the teacher-delivered didactic material and group discussion. Participants will be provided with a book on mindfulness accompanied by a guided meditation compact disc^17^. Mindfulness practice homework will begin with 5 minutes daily and will then be progressively advanced to 20 minutes daily by session 6^11^. The curriculum based research manual will be translated from English to Spanish by E.L., and a certified teacher with more than 10 years experience delivered the MAPs in English- or Spanish-speaking formats.

Health Education Program (HE)

The HE will be a weekly 2-hour, 6 session course aimed at knowledge acquisition in subjects related to health care in general. Similar interventions have been described elsewhere^11,18^. A trained health educator provided videos and didactic presentations on topics such as: stress, sleep hygiene, diet and nutrition, sexuality, mental health and substance abuse. The health education condition will resemble the MAPs intervention in terms of duration, group format and support, attention and participant expectancy regarding health benefits. Homework will include practicing health habits and weekly reading, with in-class group discussion to match the homework assigned in the MAPs group.

*Treatment Fidelity*

Therapists will be experienced and trained in one modality but not in the other. Another therapist who has extensive experience in either MAPs or HE will provide weekly supervision, and evaluated treatment integrity.

*Primary Outcome*

The primary outcome measure will be severity of depressive symptoms as assessed by the Beck Depression Inventory (BDI)^19^, with evidence that the BDI reliably evaluates depressive symptoms in non-psychiatric samples^20,21^, as well as those who are Spanish speaking^22^.

*Secondary Outcomes*

Secondary outcomes will include self-reported levels of mindfulness as measured by the Five-Facet Mindfulness Questionnaire,(FFMQ)^23^ and perceived stress, as measured by the Perceived Stress Scale^14^.

*Sample Size*

Power will be estimated in Gpower (http://www.gpower.hhu.de/en.html) and is based on previous research examining the effects of psychobehavioral interventions on depression in adults. An estimated sample size of 36 will be needed to detect a BDI between condition effect (f = 0.3, or of medium size) of difference across time based on previous MBI trials at post-intervention with 80%power^10^, two-sided P < .05, two assessment points, and 0.60 test-retest reliability for the BDI. Hence, the overall sample for this study was 72, with 36 in each language group to test between treatment effects of difference at post-intervention.

*Randomization*

Random assignment sequence will be generated via a computerized random number generator in blocks of 7 to 10 participants in MAPs and HE (1:1) for English- and Spanish speaking groups by R.O., who did not view participant data before allocation. To maintain concealment, no research staff will have access to allocation sequence, which will be recorded on sequentially numbered, opaque, and sealed envelopes.

*Blinding*

The study will be advertised as a research study to evaluate whether one or another treatment might improve perceived stress. Participants will be remain blind to hypotheses and the content of the other treatment group through study duration. Use of a modiﬁed blind-to-treatment protocol or partial blinding is thought to reduce selection bias that is frequently associated with trials of behavioral interventions. Investigators and outcome assessors will be blinded to allocation.

*Statistical analyses*

Between treatment difference in change in mean BDI at post-intervention will be the primary outcome in the intent-to-treat population. Analyses will be performed in SPSS, version 21 (IBM Corporation). Treatment effects, covarying for pre-intervention values, will be tested using a mixed model approach for primary and secondary outcomes. Data from all randomly assigned participants will be included. If missing data are less than 10%, we will impute using the expectation maximization method. The mixed model approach will generate unbiased estimates under the assumption that data are missing completely at random and the missing completely at random assumption was tested. Estimated mean differences at post-intervention will tested by treatment (MAPs vs HE) and also by language group (English vs. Spanish). Treatment effect sizes (Cohen d with Hedges bias correction for small sample size) with their 95% CIs are provided for the total sample, and for each language group.

**References**

1 Slavich, G. M. & Irwin, M. R. From stress to inflammation and major depressive disorder: a social signal transduction theory of depression. *Psychol Bull* **140**, 774-815, doi:10.1037/a0035302 (2014).

2 Kendler, K. S., Kessler, R. C., Walters, E. E., MacLean, C., Neale, M. C., Heath, A. C., Phil, D. & Eaves, L. J. Stressful life events, genetic liability, and onset of an episode of Major Depression in women. *Am J Psychiatry* **152**, 833-842 (1995).

3 Mazure, C. M. Life stressors as risk factors in depression. *Clinical Psychology: Science and Practice* **5**, 291-313 (1998).

4 Berry, J. W. in *Handbook of multicultural perspectives on stress and coping* 287-298 (Spring, 2006).

5 Romero, A. & Pina-Watson, B. in *The Oxford Handbook of Acculturation and Health* 119-130. (Oxford, 2017).

6 Nair, R. L., Roche, K. M. & White, R. M. B. Acculturation Gap Distress among Latino Youth: Prospective Links to Family Processes and Youth Depressive Symptoms, Alcohol Use, and Academic Performance. *J Youth Adolesc* **47**, 105-120, doi:10.1007/s10964-017-0753-x (2018).

7 Cobb, C. L., Xie, D., Meca, A. & Schwartz, S. J. Acculturation, discrimination, and depression among unauthorized Latinos/as in the United States. *Cultur Divers Ethnic Minor Psychol* **23**, 258-268, doi:10.1037/cdp0000118 (2017).

8 Lorenzo-Blanco, E. I., Unger, J. B., Baezconde-Garbanati, L., Ritt-Olson, A. & Soto, D. Acculturation, enculturation, and symptoms of depression in Hispanic youth: the roles of gender, Hispanic cultural values, and family functioning. *J Youth Adolesc* **41**, 1350-1365, doi:10.1007/s10964-012-9774-7 (2012).

9 Farley, T., Galves, A., Dickinson, L. M. & Perez Mde, J. Stress, coping, and health: a comparison of Mexican immigrants, Mexican-Americans, and non-Hispanic whites. *J Immigr Health* **7**, 213-220, doi:10.1007/s10903-005-3678-5 (2005).

10 Goyal, M., Singh, S., Sibinga, E. M., Gould, N. F., Rowland-Seymour, A., Sharma, R., Berger, Z., Sleicher, D., Maron, D. D., Shihab, H. M., Ranasinghe, P. D., Linn, S., Saha, S., Bass, E. B. & Haythornthwaite, J. A. Meditation programs for psychological stress and well-being: a systematic review and meta-analysis. *JAMA Intern Med* **174**, 357-368, doi:10.1001/jamainternmed.2013.13018 (2014).

11 Black, D. S., O'Reilly, G. A., Olmstead, R., Breen, E. C. & Irwin, M. R. Mindfulness meditation and improvement in sleep quality and daytime impairment among older adults with sleep disturbances: a randomized clinical trial. *JAMA Intern Med* **175**, 494-501, doi:10.1001/jamainternmed.2014.8081 (2015).

12 Brown, K. W. & Ryan, R. M. The benefits of being present: mindfulness and its role in psychological well-being. *J Pers Soc Psychol* **84**, 822-848 (2003).

13 Proulx, J., Croff, R., Oken, B., Aldwin, C. M., Fleming, C., Bergen-Cico, D., Le, T. & Noorani, M. Considerations for Research and Development of Culturally Relevant Mindfulness Interventions in American Minority Communities. *Mindfulness (N Y)* **9**, 361-370, doi:10.1007/s12671-017-0785-z (2018).

14 Cohen, S., Kamarck, T. & Mermelstein, R. A global measure of perceived stress. *J Health Soc Behav* **24**, 385-396 (1983).

15 Raison, C. L., Rutherford, R. E., Woolwine, B. J., Shuo, C., Schettler, P., Drake, D. F., Haroon, E. & Miller, A. H. A randomized controlled trial of the tumor necrosis factor antagonist infliximab for treatment-resistant depression: the role of baseline inflammatory biomarkers. *JAMA Psychiatry* **70**, 31-41, doi:10.1001/2013.jamapsychiatry.4 (2013).

16 Miller, A. H. & Raison, C. L. The role of inflammation in depression: from evolutionary imperative to modern treatment target. *Nat Rev Immunol* **16**, 22-34, doi:10.1038/nri.2015.5 (2016).

17 Smalley, S. L. & Winston, D. *Fully Present: The Science, Art, and Practice of Mindfulness*

. (Da Capo Press, 2010).

18 Irwin, M. R., Olmstead, R., Carrillo, C., Sadeghi, N., Breen, E. C., Witarama, T., Yokomizo, M., Lavretsky, H., Carroll, J. E., Motivala, S. J., Bootzin, R. & Nicassio, P. Cognitive behavioral therapy vs. Tai Chi for late life insomnia and inflammatory risk: a randomized controlled comparative efficacy trial. *Sleep* **37**, 1543-1552, doi:10.5665/sleep.4008 (2014).

19 Beck, A. T., Steer, R. A. & Garbin, M. G. Psychometric properties of the Beck Depression Inventory: Twenty-five years of evaluation. *Clinical Psychology Review* **8**, 77-100 (1988).

20 Brown, C., Schulberg, H. C. & Madonia, M. J. Assessing depression in primary care practice with the Beck Depression Inventory and the Hamilton Rating Scale for Depression. *Psychological Assessment* **7**, 59-65 (1995).

21 Bumberry, W., Oliver, J. M. & McClure, J. N. Validation of the Beck Depression Inventory in a university population using psychiatric estimate as the criterion. *Journal of Consulting and Clinical Psychology* **46**, 150-155 (1978).

22 Azocar, F., Arean, P., Miranda, J. & Munoz, R. F. Differential item functioning in a Spanish translation of the Beck Depression Inventory. *J Clin Psychol* **57**, 355-365. (2001).

23 Bohlmeijer, E., ten Klooster, P. M., Fledderus, M., Veehof, M. & Baer, R. Psychometric properties of the five facet mindfulness questionnaire in depressed adults and development of a short form. *Assessment* **18**, 308-320, doi:10.1177/1073191111408231 (2011).
